# Gene Expression Profile of Patients with Mayer-Rokitansky-Küster-Hauser Syndrome: New Insights into the Potential Role of Developmental Pathways

**DOI:** 10.1371/journal.pone.0091010

**Published:** 2014-03-07

**Authors:** Cristina Nodale, Simona Ceccarelli, Mariateresa Giuliano, Marcella Cammarota, Sirio D’Amici, Enrica Vescarelli, Diana Maffucci, Filippo Bellati, Pierluigi Benedetti Panici, Ferdinando Romano, Antonio Angeloni, Cinzia Marchese

**Affiliations:** 1 Department of Experimental Medicine, Sapienza University of Rome, Rome, Italy; 2 Department of Experimental Medicine, Biotechnology and Molecular Biology Section, Second University of Naples, Naples, Italy; 3 Department of Gynecologic-Obstetrical and Urologic Sciences, Sapienza University of Rome, Rome, Italy; 4 Department of Public Health and Infectious Diseases, Sapienza University of Rome, Rome, Italy; 5 Department of Molecular Medicine, Sapienza University of Rome, Rome, Italy; Università degli Studi di Milano, School of Medicine., Italy

## Abstract

Mayer-Rokitansky-Küster-Hauser syndrome (MRKHS) is a rare disease characterized by congenital aplasia of uterus and vagina. Although many studies have investigated several candidate genes, up to now none of them seem to be responsible for the aetiology of the syndrome. In our study, we identified differences in gene expression profile of in vitro cultured vaginal tissue of MRHKS patients using whole-genome microarray analysis. A group of eight out of sixteen MRKHS patients that underwent reconstruction of neovagina with an autologous in vitro cultured vaginal tissue were subjected to microarray analysis and compared with five healthy controls. Results obtained by array were confirmed by qRT-PCR and further extended to other eight MRKHS patients. Gene profiling of MRKHS patients delineated 275 differentially expressed genes, of which 133 downregulated and 142 upregulated. We selected six deregulated genes (*MUC1*, *HOXC8*, *HOXB2*, *HOXB5*, *JAG1* and *DLL1*) on the basis of their fold change, their differential expression in most patients and their relevant role in embryological development. All patients showed upregulation of *MUC1*, while *HOXB2* and *HOXB5* were downregulated, as well as Notch ligands *JAG1* and *DLL1* in the majority of them. Interestingly, *HOXC8* was significantly upregulated in 47% of patients, with a differential expression only in MRKHS type I patients. Taken together, our results highlighted the dysregulation of developmental genes, thus suggesting a potential alteration of networks involved in the formation of the female reproductive tract and providing a useful clue for understanding the pathophysiology of MRKHS.

## Introduction

Mayer-Rokitansky-Küster-Hauser syndrome (MRKHS) is a rare disease characterized by congenital aplasia of the uterus and the upper two-thirds of the vagina, occurring in 1 out of 4500 female births. Normal secondary sexual characteristics and karyotype (46, XX) have been shown in MRKHS women. This syndrome may occur as isolated (type I), or associated with other malformations, mainly renal, skeletal and hearing defects, and, to a lesser extent, cardiac or digital defects (type II), indicated as MURCS association (Müllerian duct aplasia, Renal dysplasia and Cervical Somite anomalies) [1], [2]. At the present, the aetiology and pathogenesis of this syndrome remain to be clarified.

The development of the female reproductive tract consists in a sequence of events governed by various transcription factors and signalling molecules. While the absence of androgens induces regression of the mesonephric duct (Wolffian duct) in females, in males the Müllerian duct degenerates through an active process mediated by the Anti-Müllerian Hormone (AMH) and its type II receptor (AMHR2) [3]. It has been proposed that inappropriate production of AMH in female embryos might induce a partial regression of the Müllerian duct, thus causing vaginal agenesis [4]. To date no mutations of *AMH* gene have been detected in MRKHS patients [5]. However, Rall *et al*. [6] hypothesized that an embryonic exposure to AMH signalling, caused by high levels of oestrogens or by deregulated expression of related genes, might occur in these patients.

MRKHS, initially considered to be sporadic, is now defined as a syndrome with autosomal-dominant inheritance with reduced penetrance and variable manifestation, consistent with a polygenic or multifactorial origin [7], [8]. However, mutations of *WNT* genes detected in MRKHS patients were indicated not responsible for the arising of the syndrome [9], [10]. Among them, heterozygous mutations of *WNT4* detected in patients with absence of uterus and vagina classify this subgroup into the so-called WNT defects for the presence of hyperandrogenism [0].

In contrast, the analysis of *HOXA* genes and hormones regulating HOX expression has yielded no mutations [13], although it has been described an hypomethylation and overexpression of *HOXA5* and *HOXA9* [6] and, in some MRKHS patients, a duplication of *SHOX*, member of the paired-related *HOX* family [14].

Interestingly, some of the malformations observed in type II MRKHS are also found in the clinical spectrum of the DiGeorge syndrome (DGS), and in DGS-like phenotypes. Consistent with these observations, whole genome analysis in MRKHS patients revealed critical chromosomal deletions in known DGS or DGS-like loci [15]–[17].

Although the genetic basis of MRKHS has been extensively investigated using different approaches, we are still far from reach a consensus on pathogenetic basis of the MRKH syndrome. The results obtained from previous studies showed a wide genetic heterogeneity among MRKHS patients. The reported discordance in monozygotic twins suggested that this syndrome might result from the combinations of transcriptional factors and epigenetic regulation other than genetic predisposition [18].

With this background, we decided to use a whole genome approach in order to identify a potentially altered gene profile in MRKHS patients. Among 2006 and 2011, we collected autologous in vitro cultured vaginal tissue from a series of 16 women with MRKHS that underwent reconstruction of neovagina and from 5 healthy controls, in order to analyze differential gene expression. Moreover, we investigated the potential relationship between gene expression profile and patients phenotype. We believe that the identification of relevant differentially expressed genes in cultured vaginal tissue may be used to better understand the molecular basis of vaginal agenesis.

## Methods

### Ethics Statements

The use of clinical samples (vaginal biopsies) for gene profiling conformed to the guidelines of the 1975 Declaration of Helsinki, as revised in 2008, and was approved by the Institutional Review Board of the Department of Gynecologic-Obstetrical and Urologic Sciences of Sapienza University of Rome. Written consent was obtained from all subjects prior to inclusion in the study. On behalf of the minors enrolled in the study, written informed consent was obtained from parents.

### Patients

We analysed sixteen women aged 16–48 years who presented utero-vaginal aplasia diagnosed by clinical examination, trans abdominal and pelvic ultrasonography, nuclear magnetic resonance (NMR) and/or vaginoscopy. All patients had a normal 46, XX karyotype. Patients underwent a check-up to search for associated malformations, including renal ultrasonography, spine radiography and echocardiography. Ten of them presented isolated utero-vaginal aplasia (MRKHS type I). The other six women displayed also other malformations, such as kidney defects (unilateral kidney agenesis, ectopic kidney) or skeletal malformations (Table 1).

**Table 1:** Summary of MRKHS patients.

Patient	Phenotype	Analysis	Genital system	Renal system	Skeleton	Heart	Hearing	Other
1	Type I	Array	Vaginal agenesis, uterushypoplasia	Normal	Normal	DiastolicDysfunctiongrade I	Normal	None
2	Type I	Array	Vaginal agenesis, uterusaplasia	Normal	Normal	Normal	Normal	Inguinal and umbilical hernia
3	Type II	Array	Vaginal agenesis, uterusaplasia	Unilateral kidney agenesis,renal ectopia	Occipital meningocele, Spina Bifida, Arnold-Chiaritype I, cifoscoliosis, C2–C3 fusion	Normal	Normal	Inguinal hernia
4	Type II	Array	Rudimentary uterus,vaginal agenesis	Unilateral kidneyagenesis	Normal	Normal	Normal	None
5	Type I	Array	Vaginal agenesis,rudimentary uterus	Normal	Normal	Normal	Normal	None
6	Type I	Array	Unilateralsalpingectomy	Normal	Normal	Normal	Not determined	None
7	Type I	Array	Vaginal agenesis,uterus aplasia	Normal	Normal	Normal	Normal	None
8	Type II	Array	Vaginal agenesis	Normal	Normal	Normal	Normal	None
9	Type I	qRT-PCR	Vaginal agenesis	Normal	Normal	Normal	Normal	None
10	Type I	qRT-PCR	Vaginal atresia,uterus aplasia	Normal	Normal	Normal	Normal	Inguinal hernia
11	Type I	qRT-PCR	Rudimentary uterus,vaginal agenesis	Normal	Normal	Normal	Normal	Notta’s syndrome
12	Type I	qRT-PCR	Vaginal agenesis	Normal	Normal	Normal	Normal	None
13	Type I	qRT-PCR	Vaginal agenesis	Normal	Normal	Normal	Normal	None
14	Type II	qRT-PCR	Vaginal agenesis,uterus aplasia	Ectopic kidney	Normal	Dextrocardia	Normal	Unilateral lung agenesis, cleft lip and palate
15	Type I	qRT-PCR	Vaginal agenesis	Normal	Normal	Normal	Notdetermined	None
16	Type II	qRT-PCR	Half-uterus, unilateralhematosalpinx	Unilateral kidneyagenesis	Normal	Interventricularseptal defect	Notdetermined	Oesophageal atresia

Patients were analysed for follicle-stimulating hormone (FSH), luteinizing hormone (LH), 17 beta-estradiol (E2), dehydroepiandrosterone sulphate (DHEAS), progesterone and testosterone. All hormonal indicators were consistent with physiological levels with rispect to age of the patients.

Samples obtained from vaginal tissue biopsy of eight out of sixteen patients (mean age 24.0 years: range 16–48 years) were subjected to microarray analysis, whereas samples from all patients (mean age 23.7 years: range 16–48 years) were analysed through quantitative Real Time PCR (qRT-PCR). Specimens from five healthy women (mean age 31.8 years: range 27–40 years) were used as control.

### Cell Cultures

Primary cultures of human vaginal mucosa cells (HVMs) were established from 1 cm^2^ full-thickness mucosal biopsy of the vaginal vestibule of MRKHS patients and vagina of healthy controls. Following enzymatic dissociation, cells were seeded onto collagen IV (10 mg/ml)-coated culture plates and maintained in chemical defined Keratinocyte Growth Medium (KGM; Lonza Milano S.r.l., Milan, Italy), with medium change twice a week.

Primary culture of buccal mucosa cells from one MRKHS patient enrolled in this study was also obtained with the same procedure.

Cell cultures were examined by immunofluorescence and western blot analysis as described in Data S1 and their morphology was evaluated with a phase contrast microscopy. All cell cultures consisted of epithelial cells as they exhibited the cobblestone-like appearance. Moreover the expression of specific epithelial markers (K14 and K19) and lack of vimentin confirmed their epithelial origin (Fig. S1).

### RNA Preparation

Total RNA from cultures derived from vaginal and buccal mucosa was extracted using TRIzol reagent (Invitrogen, Milan, Italy) following the manufacturer’s instructions. RNA samples were quantified using a NanoDrop ND-1000 spectrophotometer (NanoDrop, Wilmington, DE, USA) and evaluated for degradation using an Agilent 2100 Bioanalyzer (Agilent Technologies, Santa Clara, CA, USA). The samples were selected for gene expression array analysis on the basis of compliance to the following criteria: RIN value >7, absorption ratio 260∶280 ≥1.9, and ratio of the ribosomal bands 28S∶18S >1.5.

### Gene Expression Assay

Expression profiling was accomplished using the HumanHT-12 v3 whole-genome gene expression direct hybridization assay (Illumina, Inc., San Diego, CA, USA), with six arrays on each single BeadChip. Each array is comprised of >48,000 probes derived from human genes in the NCBI RefSeq and UniGene databases. A total of thirteen separate samples (eight patients and five healthy controls) were interrogated on the BeadChip arrays, with no pooling of samples within groups, in order to evaluate sample-to-sample variability in gene expression both within and across each group.

Initially, 100 ng of total RNA was converted to cDNA, followed by an in vitro transcription step to generate labelled cRNA using the Ambion Illumina TotalPrep RNA Amplification Kit (Ambion, Austin, TX, USA) according to the manufacturer’s instructions. The labelled probes were then mixed with hybridization reagents and hybridized overnight to the HumanHT-12 v3.0 BeadChips. Following washing and staining, the BeadChips were imaged using the Illumina BeadArray Reader to measure fluorescence intensity at each probe. The intensity of the signal corresponded to the amount of the respective mRNA in the original sample.

### Microarray Expression Analysis

BeadChip data files were analysed with Illumina’s GenomeStudio gene expression module (and Bioconductor package) to determine gene expression signal levels. Briefly, the raw intensity of Illumina Human HT-12 v3.0 gene expression array was scanned and extracted using BeadScan, with the data corrected by background subtraction in GenomeStudio module.

The data obtained from the two populations were analysed for differences in expression. Up or down regulated transcript lists were obtained using average normalization, error model Illumina Custom, selected by p<0.00001, Diff Score>20 and fold change >2.

Heatmap was generated through the web interface Matrix2png, version 1.2.2 (http://www.chibi.ubc.ca/matrix2png/) [19].

Area-proportional Venn diagram for the comparison and visualization of biological lists of patients affected by type I or type II MRKHS was generated through the web application BioVenn (http://www.cmbi.ru.nl/cdd/biovenn/) [20].

The data discussed in this publication have been deposited in NCBI’ s Gene Expression Omnibus and are accessible through GEO Series accession number GSE52609 (http://www.ncbi.nlm.nih.gov/geo/query/acc.cgi?acc=GSE52609).

### Pathway Analysis

Differentially expressed genes were subjected to systematic network analysis to determine the biological processes and pathways associated with each gene using WebGestalt (WEB-based GEne SeT AnaLysis Toolkit) (http://bioinfo.vanderbilt.edu/webgestalt/, Nashville, TN). Only genes that were significantly increased or decreased were included in the pathway analysis. The assignment of all differentially expressed genes to functional groups was performed through the Gene Ontology Slim Biological Process classification and reported as a graph.

### Quantitative Real Time PCR (qRT-PCR)

1µg of total RNA was reverse transcribed using High Capacity RNA to cDNA Kit (Applied Biosystems by Life Technologies, Carlsbad, CA, USA) according to the manufacturer’s instructions. cDNA was diluted 1∶5 and then used for amplification of *MUC1*, *HOXC8*, *HOXB2*, *HOXB5*, *JAG1* and *DLL1* using the appropriate TaqMan gene expression assay kits (Applied Biosystems). A total of 2 µl/well of template was added to the sample wells along with Taqman Universal PCR master mix at a concentration of 1x and water to a volume of 25 µl/well. Assays were conducted in triplicate on an ABI 7500 Real Time instrument (Applied Biosystems) using the following conditions: 50°C for 2 min, 95°C for 10 min, and then 95°C for 15 s and 60°C for 1 min, repeated 40 times. Relative quantification was performed using *GAPDH* mRNA as an endogenous control.

### Statistical Methods

Statistical analysis for significance was performed using Student *t*-test. *P* values less than 0.05 were considered statistically significant.

## Results

### Cluster Analysis and Identification of Differentially Expressed Genes

**Figure 1:**
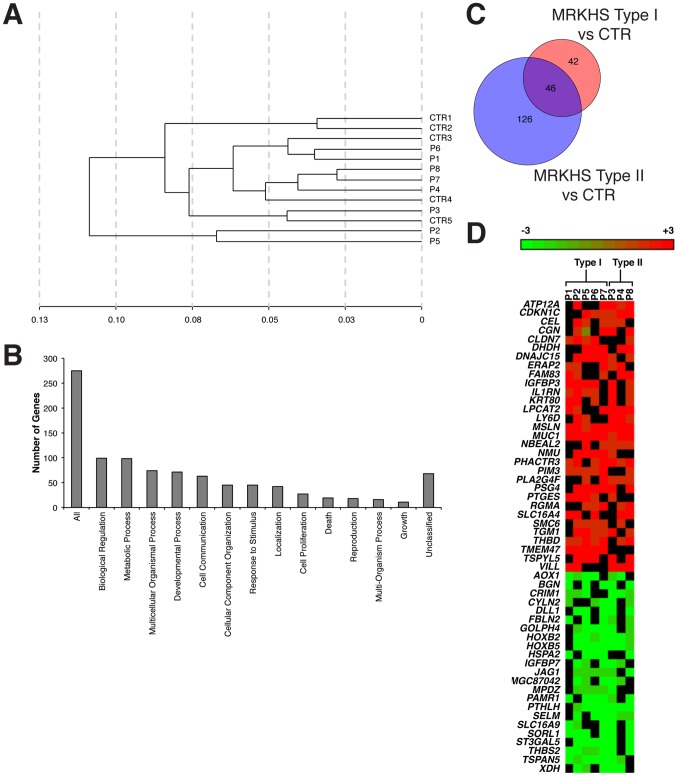
Gene expression profile of MRKHS patients. **A**) Clustering analysis of gene expression profiles of MRKHS patients and healthy controls. The distance on the X-axis represents the similarity relationships among samples. The label CTR (1–5) represents for healthy controls, the label P (1–8) represents for MRKHS patients. **B**) Assignment of differentially expressed genes to biological process categories. The y-axis indicates the number of differentially expressed genes within each biological process category. **C**) Venn diagram of similarities and differences in type I and type II MRKHS profiles. Each of the circles depicts the number of different transcripts identified as statistically significant in the comparison of the two sample groups (type I, red; type II, blue) with healthy control (CTR). Overlapping differences shared among the two sample groups are represented in the area of intersection between the two circles (violet). **D**) Heatmap of the expression profile of each MRKHS patient. The cut-off for inclusion in the heatmap was the alteration of gene expression in more than half of patients.

Total RNA obtained by cell cultures derived from vaginal mucosa of eight MRKHS patients, five with type I MRKHS and three with type II MRKHS (Table 1, patients numbered from 1 to 8), and from vaginal mucosa of five healthy controls were analysed by microarray. The hierarchical clustering analysis (Fig. 1A) revealed that only two out of five control RNAs (CTR1–5) formed an independent cluster away from the patients groups, while the other controls were distributed within the three sub-clusters formed by most of patients’ samples. Two of the eight patients (P2 and P5) formed an independent cluster away from the others, which formed three sub-clusters (P1 and P6; P4, P7 and P8; P3) related each other with subsequently decreased similarity. This pattern indicated that patients were heterogeneous and therefore this might be potentially due to the multifactorial aetiology of the syndrome.

To identify differentially expressed genes in a statistically significant manner, we performed fold-change data analysis. The gene list obtained from microarray was filtered to identify candidate genes for which the expression levels differed by at least 2 fold between the two groups. After statistical testing procedures, removal of the transcripts with no Entrez gene ID and fold change cut-off (Fold≥2), we identified a total of 277 differentially expressed genes of which 133 downregulated and 142 upregulated.

The differentially expressed genes in MRKHS patients were then categorized by biological process. A large proportion of genes were mainly involved in functions relevant to tissue patterning and organogenesis, cell proliferation/differentiation, cell communication, cellular motility, endocrine and reproductive disorders, and others (Fig. 1B).

Pairwise comparisons between type I and type II groups were carried out, differentially expressed genes were identified for each comparison and are illustrated in a Venn diagram (Fig. 1C). The higher number of differentially expressed genes, detected in the comparison of the type II group versus CTR with respect to the type I group versus CTR (172 and 88 genes, respectively), was consistent with the severity of type II MRKHS, which clinical features imply the involvement of a higher number of organs and systems.

The overlapping region (violet) contained 46 transcripts that were significantly different in both the comparisons, thus indicating their potential role in MRKHS aetiology. These genes are listed in Table 2.

**Table 2:** List of 46 genes showing altered expression in both type I and type II MRKHS.

Probe set ID	Gene title	Gene symbol
ILMN_1780806	Homo sapiens ankyrin repeat domain 36B (ANKRD36B), mRNA.	*ANKRD36B*
ILMN_1767113	Homo sapiens aldehyde oxidase 1 (AOX1), mRNA.	*AOX1*
ILMN_1656248	Homo sapiens adipocyte-specific adhesion molecule (ASAM), mRNA.	*ASAM*
ILMN_2206746	Homo sapiens biglycan (BGN), mRNA.	*BGN*
ILMN_2341611	Homo sapiens CUB domain containing protein 1 (CDCP1), transcript variant 2, mRNA.	*CDCP1*
ILMN_2146418	Homo sapiens cysteine rich transmembrane BMP regulator 1 (chordin-like) (CRIM1), mRNA.	*CRIM1*
ILMN_1689200	Homo sapiens dihydrodiol dehydrogenase (dimeric) (DHDH), mRNA.	*DHDH*
ILMN_1743373	Homo sapiens delta-like 1 (Drosophila) (DLL1), mRNA.	*DLL1*
ILMN_1812666	Homo sapiens DnaJ (Hsp40) homolog, subfamily C, member 15 (DNAJC15), mRNA.	*DNAJC15*
ILMN_1677466	Homo sapiens dual specificity phosphatase 6 (DUSP6), transcript variant 1, mRNA.	*DUSP6*
ILMN_1743145	Homo sapiens endoplasmic reticulum aminopeptidase 2 (ERAP2), mRNA.	*ERAP2*
ILMN_1774602	Homo sapiens fibulin 2 (FBLN2), transcript variant 2, mRNA.	*FBLN2*
ILMN_1727992	Homo sapiens fasciculation and elongation protein zeta 1 (zygin I) (FEZ1), transcript variant 2, mRNA.	*FEZ1*
ILMN_1768260	Homo sapiens growth arrest-specific 6 (GAS6), mRNA.	*GAS6*
ILMN_1795344	Homo sapiens golgi phosphoprotein 4 (GOLPH4), mRNA.	*GOLPH4*
ILMN_1810274	Homo sapiens homeobox B2 (HOXB2), mRNA.	*HOXB2*
ILMN_1674908	Homo sapiens homeobox B5 (HOXB5), mRNA.	*HOXB5*
ILMN_1746085	Homo sapiens insulin-like growth factor binding protein 3 (IGFBP3), transcript variant 2, mRNA.	*IGFBP3*
ILMN_2062468	Homo sapiens insulin-like growth factor binding protein 7 (IGFBP7), mRNA.	*IGFBP7*
ILMN_1774874	Homo sapiens interleukin 1 receptor antagonist (IL1RN), transcript variant 4, mRNA.	*IL1RN*
ILMN_1691376	Homo sapiens jagged 1 (Alagille syndrome) (JAG1), mRNA.	*JAG1*
ILMN_1705814	Homo sapiens keratin 80 (KRT80), transcript variant 1, mRNA.	*KRT80*
ILMN_3236021	PREDICTED: Homo sapiens hypothetical protein LOC100133923 (LOC100133923), mRNA.	*LOC100133923*
ILMN_1725750	PREDICTED: Homo sapiens hypothetical LOC644695 (LOC644695), mRNA.	*LOC644695*
ILMN_3200140	PREDICTED: Homo sapiens misc_RNA (LOC645638), miscRNA.	*LOC645638*
ILMN_1694778	PREDICTED: Homo sapiens similar to Keratin, type I cytoskeletal 18 (Cytokeratin-18) (CK-18) (Keratin-18) (K18) (LOC646723), mRNA.	*LOC646723*
ILMN_1697377	PREDICTED: Homo sapiens similar to protein immuno-reactive with anti-PTH polyclonal antibodies (LOC649841), mRNA.	*LOC649841*
ILMN_1667932	PREDICTED: Homo sapiens similar to ankyrin repeat domain 36 (LOC652726), mRNA.	*LOC652726*
ILMN_1663575	PREDICTED: Homo sapiens similar to Six transmembrane epithelial antigen of prostate (MGC87042), mRNA.	*MGC87042*
ILMN_2353161	Homo sapiens mesothelin (MSLN), transcript variant 2, mRNA.	*MSLN*
ILMN_1677314	Homo sapiens mucin 1, cell surface associated (MUC1), transcript variant 1, mRNA.	*MUC1*
NM_152673.1	Homo sapiens mucin 20, cell surface associated (MUC20), transcript variant S, mRNA.	*MUC20*
ILMN_1658356	Homo sapiens peptidase domain containing associated with muscle regeneration 1 (PAMR1), transcript variant 1, mRNA.	*PAMR1*
NM_080672.3	Homo sapiens phosphatase and actin regulator 3 (PHACTR3), transcript variant 3, mRNA.	*PHACTR3*
ILMN_2384745	Homo sapiens pregnancy specific beta-1-glycoprotein 4 (PSG4), transcript variant 1, mRNA.	*PSG4*
ILMN_1785699	Homo sapiens parathyroid hormone-like hormone (PTHLH), transcript variant 3, mRNA.	*PTHLH*
ILMN_1744210	Homo sapiens succinate dehydrogenase complex, subunit A, flavoprotein (Fp) (SDHA), nuclear gene encoding mitochondrialprotein, mRNA.	*SDHA*
ILMN_1651429	Homo sapiens selenoprotein M (SELM), mRNA.	*SELM*
ILMN_1732410	Homo sapiens solute carrier family 16, member 9 (monocarboxylic acid transporter 9) (SLC16A9), mRNA.	*SLC16A9*
ILMN_2060115	Homo sapiens sortilin-related receptor, L(DLR class) A repeats-containing (SORL1), mRNA.	*SORL1*
ILMN_1713496	Homo sapiens ST3 beta-galactoside alpha-2,3-sialyltransferase 5 (ST3GAL5), transcript variant 2, mRNA.	*ST3GAL5*
NM_004605.2	Homo sapiens sulfotransferase family, cytosolic, 2B, member 1 (SULT2B1), transcript variant 1, mRNA.	*SULTB1*
NM_000359.1	Homo sapiens transglutaminase 1 (K polypeptide epidermal type I, protein-glutamine-gamma-glutamyltransferase) (TGM1),mRNA.	*TGM1*
ILMN_1759787	Homo sapiens thrombomodulin (THBD), mRNA.	*THBD*
ILMN_1799028	Homo sapiens tetraspanin 5 (TSPAN5), mRNA.	*TSPAN5*
NM_145652.2	Homo sapiens WAP four-disulfide core domain 5 (WFDC5), mRNA.	*WFDC5*

Heat map was provided to visualize the differentially expressed genes in each patient (Fig. 1D). We considered only genes deregulated in more than half of patients (>4).

Among the deregulated genes, we analysed *HOX* family in detail. MRKHS patients showed downregulation of *HOXA13* (−2.0 fold), *HOXB2* (−3.4 fold), *HOXB4* (−4.2 fold), *HOXB5* (−4.1 fold) and *HOXB7* (−4.2 fold), and upregulation of *HOXC6* (6.8 fold) and *HOXC8* (38.2 fold). In particular we focused on *HOXB2* and *HOXB5*, since they were included in the list of type I and type II overlapping genes (Table 2) and they were highly decreased in seven out of eight patients (Fig. 1D). *HOXC8* also came to our attention despite the fact that it was altered in just three out of eight patients (Fig. 1D), since its overexpression was particularly high (38.2 fold increase). Characteristically, its deregulation was observed only in type I MRKHS patients.

We found also interesting the downregulation of *JAG1* and *DLL1* genes, encoding for Notch ligands. They were significantly decreased (−3.4 fold and −2.2 fold, respectively) in six and five out of eight patients, respectively, and in both type I and type II MRKHS patients; based upon these findings and their role in development we decided to further investigate them.

Among overexpressed genes, *MUC1* and *MSLN*, coding for glycoproteins, were found to be significantly upregulated in all patients analysed by microarray (6.7 and 4.2 fold, respectively) (Fig. 1D). We focused in particular on MUC1, which is known to have a key role in the stabilization of β-catenin, an essential mediator of canonical Wnt signalling involved in female tract development [21], [22].

### Validation of Array-based Gene Expression Profiles by Quantitative Real Time PCR (qRT-PCR)

qRT-PCR validation of differentially expressed mRNA was performed on all MRKHS patients tested for microarray analysis. Six genes (*MUC1*, *HOXC8*, *HOXB2*, *HOXB5*, *JAG1*, *DLL1*) were selected on the basis of their fold change, their differential expression in the majority of patients and/or their embryologically relevant role in the development of the female genital tract (Table 3).

**Table 3:** Genes selected for qRT-PCR validation based on altered expression in microarray assay.

Probe set ID	Gene title	Genesymbol	Biological role	Fold change	N. patients
ILMN_1677314	Mucin 1, cell surface associated	*MUC1*	ECM-cell interaction, IL-7 signalling pathway	6.7	8/8
ILMN_1718285	Homeobox C8	*HOXC8*	System and cellular development, organ morphogenesis	38.2	3/8
ILMN_1810274	Homeobox B2	*HOXB2*	Embryonic and adult development, epithelial morphogenesis	−3.4	7/8
ILMN_1674908	Homeobox B5	*HOXB5*	Embryonic and adult development, epithelial morphogenesis	−4.1	7/8
ILMN_1743373	Delta-like 1 (Drosophila)	*DLL1*	Notch pathway, organ and cellular development, cell adhesion,cell differentiation	−3.4	5/8
ILMN_1691376	Jagged 1 (Alagille syndrome)	*JAG1*	Notch pathway, organ and cellular development, epithelialmorphogenesis, cell differentiation and proliferation	−2.2	6/8

**Figure 2:**
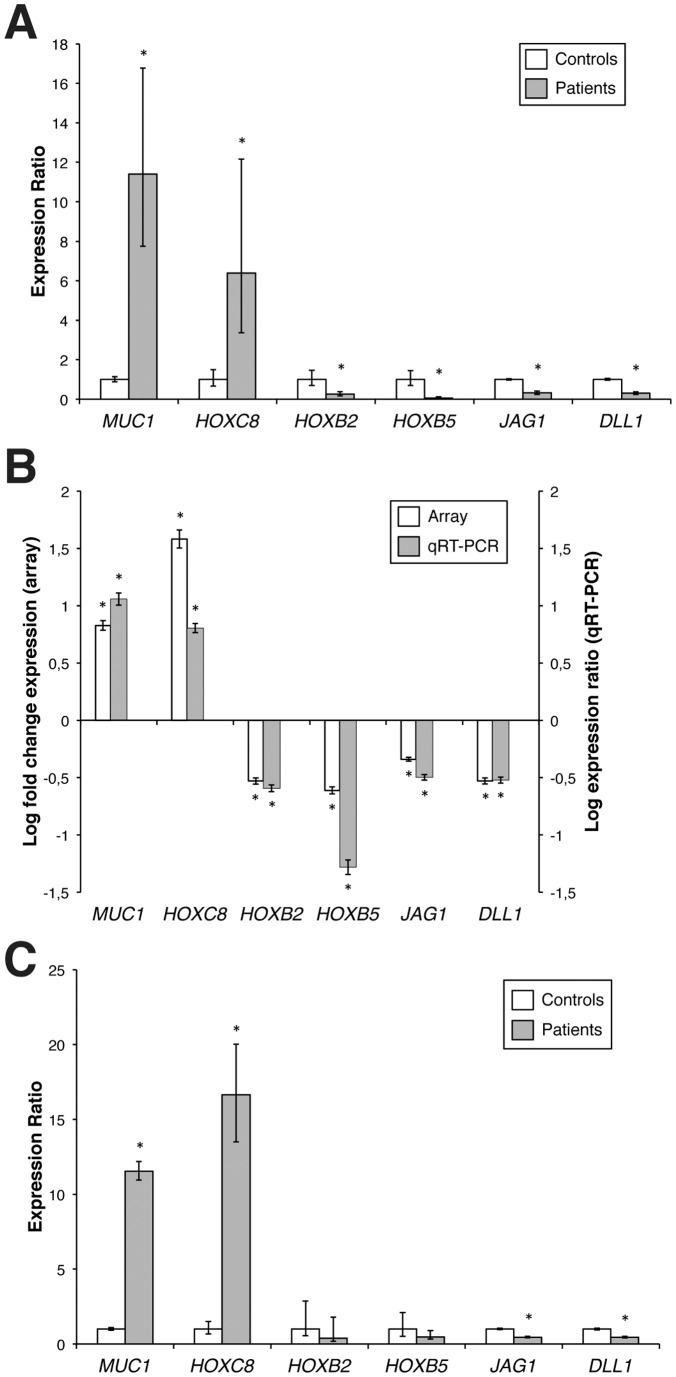
Validation of differential gene expression by Quantitative Real Time PCR. **A**) qRT-PCR analysis of mRNA expression levels of the six selected genes in the eight patients subjected to microarray analysis. For each gene, relative mRNA levels of patients are shown as fold value of the levels of five healthy subjects (controls). Each experiment was performed in triplicate, and mRNA levels were normalized to *GAPDH* mRNA expression. Error bars represent standard deviations (*P<0.01). **B**) Expression of selected genes in MRKHS patients compared to controls, tested by microarray analysis (left, white columns) and qRT-PCR (right, grey columns). Array data are shown as log fold change of patients divided by controls, qRT-PCR data are shown as log expression ratio of patients divided by controls. Bars indicate the measurement error (*P<0.01). **C**) qRT-PCR analysis of mRNA expression levels of the genes of interest in sixteen MRKHS patients. For each gene, relative mRNA levels of patients are shown as fold value of the levels of five healthy subjects (controls). Each experiment was performed in triplicate, and mRNA levels were normalized to *GAPDH* mRNA expression. Error bars represent standard deviations (*P<0.01).

All selected genes were confirmed to have significant alterations in their expression in MRKHS patients; *MUC1* and *HOXC8* were strongly upregulated (11.4 and 6.4 fold, respectively, *P*<0.01), while *HOXB2*, *HOXB5*, *JAG1* and *DLL1* were downregulated (0.26, 0.05, 0.32 and 0.30 fold, respectively, *P*<0.01) (Fig. 2A). Moreover, qRT-PCR results (Fig. 2B, grey columns) showed 100% validation efficiency in comparison to the expression data of microarray experiments (Fig. 2B, white columns).

Therefore, qRT-PCR results validated the microarray findings, thus confirming the modulation of all selected genes in the eight MRKHS patients.

### qRT-PCR in an Extended Cohort of MRKHS Patients

Expression analysis of the six selected genes was then conducted in an extended cohort of patients, comprising other eight MRKHS patients, five with type I MRKHS and three with type II MRKHS (Table 1, patients numbered from 9 to 17). We performed qRT-PCR analysis of sixteen patients in order to verify the trend of selected genes (Fig. 2C).

In the total cohort of patients, *MUC1* and *HOXC8* mRNA levels resulted to be significantly overexpressed (11.7 and 13.7 fold, respectively, *P*<0.01). The hypothesis of an involvement of MUC1 in the pathogenesis of MRKHS is sustained by the observation that all patients (100%) showed upregulation of this mucin through microarray analysis, and fifteen out of sixteen MRKHS patients (93.8%) showed a significant overexpression of this gene also by qRT-PCR. In the case of *HOXB2* and *HOXB5*, we confirmed the decreasing trend with respect to healthy controls (0.42 and 0.49 fold, respectively), despite their very low basal expression levels.

As concerning Notch ligands, both *JAG1* and *DLL1* were confirmed to be significantly downregulated (0.43 and 0.44 fold, *P*<0.01) also extending the number of MRKHS patients.

Highly significant downregulation (*P*<0.05) of *JAG1* was observed in a total of ten MRKHS patients (62.5%), while *DLL1* expression was significantly deregulated in all patients (100%). These data pointed out a strong role of Notch signalling in MRKHS pathogenesis.

*HOXC8* was significantly upregulated (*P*<0.05) in seven out of sixteen MRKHS patients (47%) (Fig. 2C). These patients showed very high levels of *HOXC8* mRNA, ranged from 3.8 to 47.1 fold.

Data obtained from qRT-PCR from buccal mucosa of one MRKHS patient confirmed this trend indicating that alterations in the gene expression are not only confined to cells derived from vaginal mucosa but are also extended to other tissues although with a lower fold increase and limited sample number (Fig. S2).

**Figure 3:**
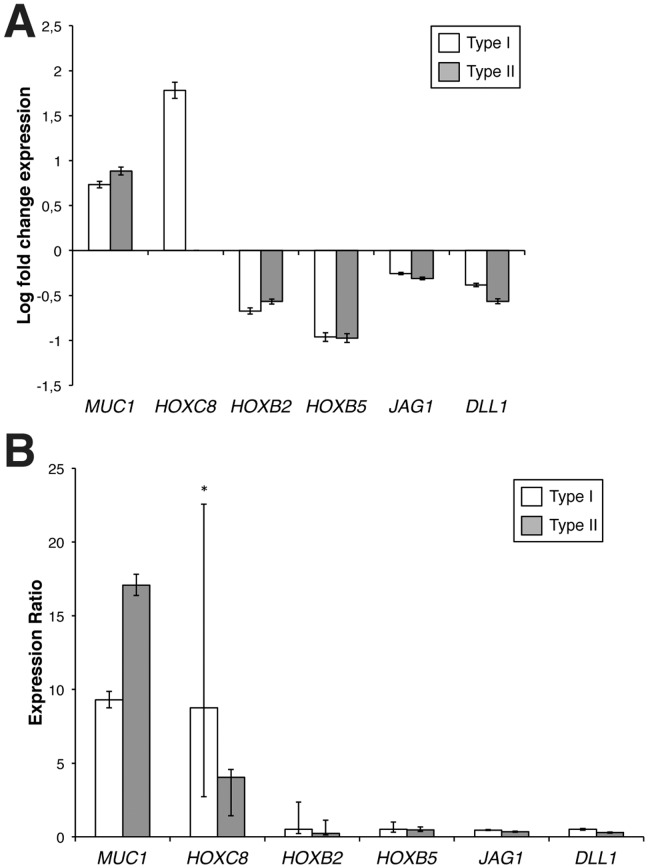
Expression levels of the genes of interest in type I and type II MRKHS patients. **A**) Expression of selected genes in type I (white columns) and type II (grey columns) MRKHS patients tested by microarray analysis. Array data are shown as log fold change of patients divided by controls. Bars indicate the measurement error (*P<0.01). **B**) qRT-PCR analysis of mRNA expression levels of the selected genes in type I (white columns) and type II (grey columns) MRKHS patients. For each gene, relative mRNA levels of patients are shown as fold value of the levels of five healthy subjects (controls). Each experiment was performed in triplicate, and mRNA levels were normalized to *GAPDH*.

Finally, we compared the transcript levels of the analysed genes between patients with type I and type II MRKHS. Microarray analysis pointed out the existence of a series of 46 genes differentially expressed in both type I and type II MRKHS patients, as reported in Fig. 1C and Table 2. Five out of the six selected genes were included in this category, and their fold change with respect to healthy controls did not significantly differ in the two groups (Fig. 3A). As concerning *HOXC8*, however, microarray data indicated a differential expression of this gene only in type I MRKHS patients (Fig. 3A). Data obtained from qRT-PCR on all patients (ten type I and six type II MRKHS) confirmed this trend, with significantly different expression levels between the two groups (Fig. 3B).

## Discussion

During embryogenesis, specific signals are required to regulate proper female tract development different genes involved in such signals have been proposed as candidates responsible for the arising of MRKHS. At the present, however, the aetiology of this syndrome is still poorly understood and MRKHS is considered a multifactorial disorder.

Several studies have investigated mutations in developmental genes, without being able to prove a direct link with the onset of MRKHS [5], [9]–[12]. Recent findings based upon discordance between monozygotic twins suggested the involvement of epigenetic factors [18]. In this context, the analysis of gene expression became a feasible approach, even if it could be performed only on adult tissues.

A recent study analysed gene expression profile of uterine tissue of MRKHS patients [6]. Combining whole-genome and methylation arrays, the authors detected a series of hypermethylated and downmodulated or hypomethylated and overexpressed genes.

In the present study, we considered an alternative approach, carrying out for the first time gene expression profiling on cultured cells harvested from vaginal vestibulum biopsy avoiding the use of uterine or other derivation tissues in order to place all samples in the same conditions.

We identified 275 differentially expressed genes, 142 downregulated and 133 upregulated. Among them, we focused our attention on six genes (*MUC1*, *HOXC8*, *HOXB2*, *HOXB5*, *JAG1*, *DLL1*).

*MUC1*, a heavily glycosylated transmembrane protein expressed in most of secretory epithelia, resulted overexpressed in all MRKHS patients analysed by microarray. Moreover, *MUC1* mRNA has been found significantly deregulated in 93.4% of MRKHS patients assessed by qRT-PCR analysis. These results suggested for the first time the potential involvement of a mucin in the pathogenesis of MRKHS.

Several studies showed thatthe cytoplasmic domain of MUC1 specifically interacts with β-catenin [21], [22], an essential mediator of canonical Wnt signalling that plays a critical role in female tract development through its direct and indirect action on AMH and its receptor [23], [24]. Since MUC1 overexpression stabilizes β-catenin and enhances its nuclear translocation [21], we hypothesized that its overexpression during foetal development might induce improper AMH-AMHR2 activation, thus causing a partial Müllerian duct regression.

Our results pointed out also a potential alteration of Notch signalling pathway through the downregulation of its specific ligands *JAG1* and *DLL1*. In particular, we found deregulated *JAG1* in a high percentage of MRKHS patients (62.5%), and *DLL1* even in all patients (100%). It is known that Notch signalling influences differentiation of many tissues, such as heart and kidney [25], but its potential role in the development of the reproductive tract has never been fully investigated. Turner *et al*. [26] proposed a role for Notch in normal testosterone-mediated development of the Wolffian duct. In humans, mutations in the *JAG1* gene cause the Alagille syndrome, characterized by severe cardiac malformation and developmental anomalies in several organs, including kidney [27], [28]. Perturbations in Notch signalling also contribute to the aetiology of Klippel-Feil syndrome [29] and spondylocostal dysostosis [30], skeletal disorders often observed in association with vaginal agenesis [31], [32]. Such data led us to hypothesize a role for this gene family in the aetiopathogenesis of type II MRKHS. Nevertheless, we did not observe any statistically significant difference in *JAG1* or *DLL1* expression among type I and type II MRKHS patients. Notch ligands deregulation presumably acts in concert with genetic background, other specific gene mutation-dysregulation and/or developmental insults in the pathogenesis of MRKHS. The crosstalk between Notch and Wnt pathways has been described in different types of tumour cells [33] and in intestinal epithelial cell fate decision [34]. Similar interactions might be also required in the development of female reproductive organs. Although no mutations were found in *WNT* gene family in previous studies, it is conceivable that alteration in Notch pathways might also affect Wnt pathway. In this regard, it has been reported that Notch1 counteracts Wnt/β-catenin signalling through epigenetic modifications [35]. Interestingly, our microarray analysis revealed a downregulation of *WNT5A*, *WNT7A* and *WNT7B* in some MRKHS patients (data not shown).

Consistently with a previously published microarray study on MRKHS patients in which Rall *et al*. [6] reported a deregulation of *HOXA* genes, we observed a significant downregulation of *HOXB2* and *HOXB5* in most of MRKHS patients. At the same time, we observed a strong overexpression of *HOXC8*, which is known to be involved in correct patterning of the axial skeleton along the antero-posterior axis during early embryogenesis [36] and in kidney differentiation [37], only in type I MRKHS patients. In light of these considerations, we can hypothesize that a consistent overexpression of *HOXC8* might be a compensatory mechanism to offset the decreased expression of other developmental genes, which can limit the onset of other malformations, especially those involving renal system, and then produce a milder phenotype (type I MRKHS).

Taken together, our results suggest a potential dysregulation of a network of developmental genes that may disrupt Müllerian duct formation. Those genes may represent key players that, acting in a complex network, contribute to the arising and pathogenesis of MRKHS. Future studies will contribute to a better understanding of the biological role of these factors in this syndrome.

## Supporting Information

Figure S1 Characterization of primary cell cultures obtained from vaginal biopsy (HVMs). **A**) Representative morphology of cell cultures. Images were assessed by phase contrast microscopy. Scale bar 100 µm. **B**) Representative immunofluorescence expression of K14 and K19 in HVMs. Scale bar 100 µm. **C**) Representative western blot analysis of K14, K19 and Vimentin in HVMs. HeLa, MCF7 and HF were used as positive or negative control. Anti-Tubulin antibody was used as loading control. The images are representative of at least three independent experiments.
(TIF)

Figure S2 Comparison of expression levels of selected genes in vaginal mucosa and buccal mucosa of one MRKH patients. **A**) qRT-PCR analysis of mRNA expression levels of the six selected genes, in cell culture from vaginal mucosa of one MRKHS patient. For each gene, relative mRNA levels of patients are shown as fold value of the levels of one healthy subject (control). Each experiment was performed in triplicate, and mRNA levels were normalized to GAPDH mRNA expression. Error bars represent standard deviations (*P<0.01). **B**) qRT-PCR analysis of mRNA expression levels of the six selected genes in cell culture from buccal mucosa of one MRKHS patient. For each gene, relative mRNA levels of patient are shown as fold value of the levels of one healthy subject (control). Each experiment was performed in triplicate, and mRNA levels were normalized to GAPDH mRNA expression. Error bars represent standard deviations (*P<0.01). mRNA expression. Error bars represent standard deviations (*P<0.01).
(TIF)

Data S1 Supplementary Material and Methods.
(DOC)
