# Supplementary figures and images for: Gene Expression Profile of Patients with Mayer-Rokitansky-Küster-Hauser Syndrome: New Insights into the Potential Role of Developmental Pathways
Source: PLoS One. 2014 Mar 7;9(3):e91010. doi: 10.1371/journal.pone.0091010 (PMC3946625; doi:10.1371/journal.pone.0091010)

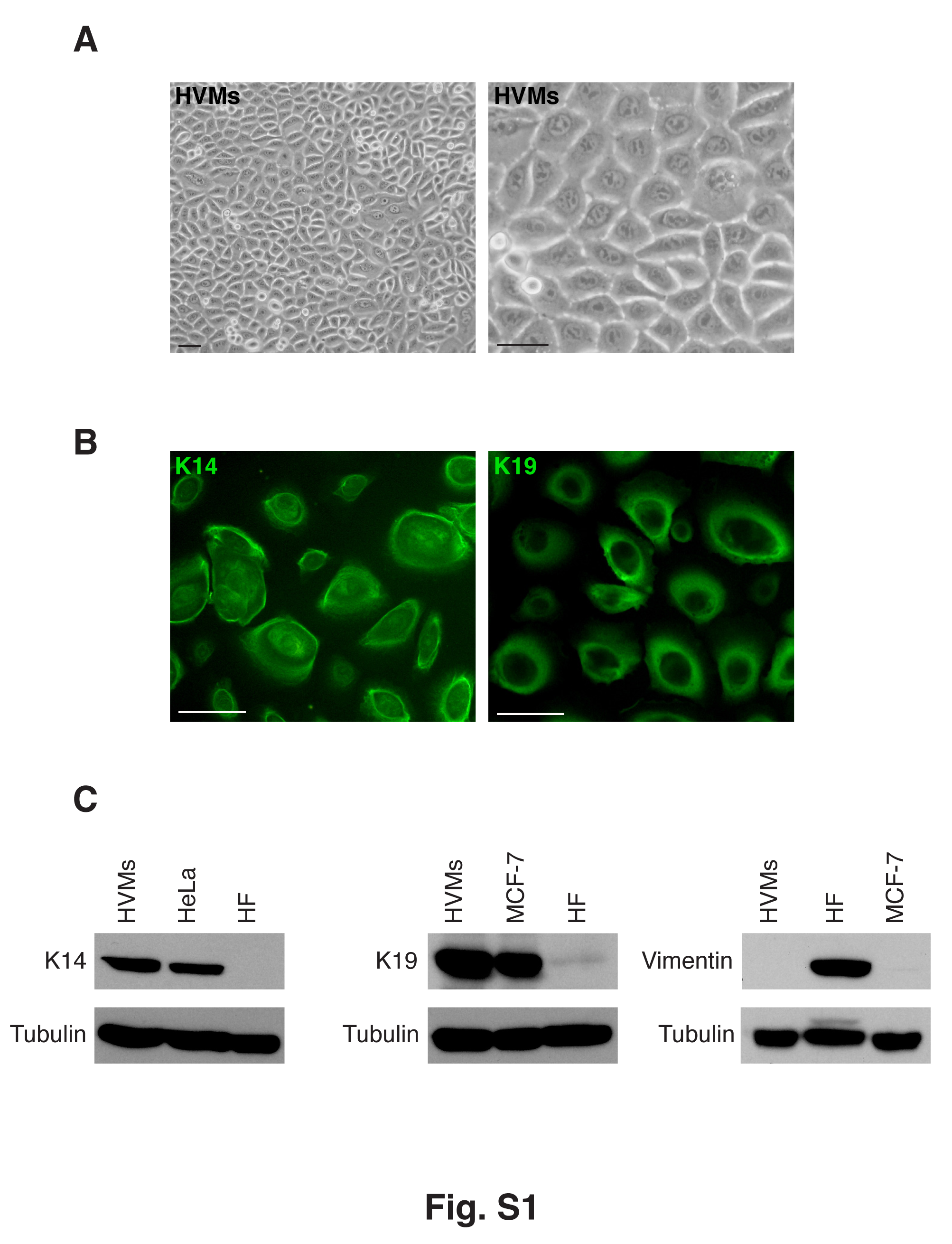

Supplement: Figure S1 — Characterization of primary cell cultures obtained from vaginal biopsy (HVMs). A) Representative morphology of cell cultures. Images were assessed by phase contrast microscopy. Scale bar 100 µm. B) Representative immunofluorescence expression of K14 and K19 in HVMs. Scale bar 100 µm. C) Representative western blot analysis of K14, K19 and Vimentin in HVMs. HeLa, MCF7 and HF were used as positive or negative control. Anti-Tubulin antibody was used as loading control. The images are representative of at least three independent experiments. (TIF) [file pone.0091010.s001.tif]

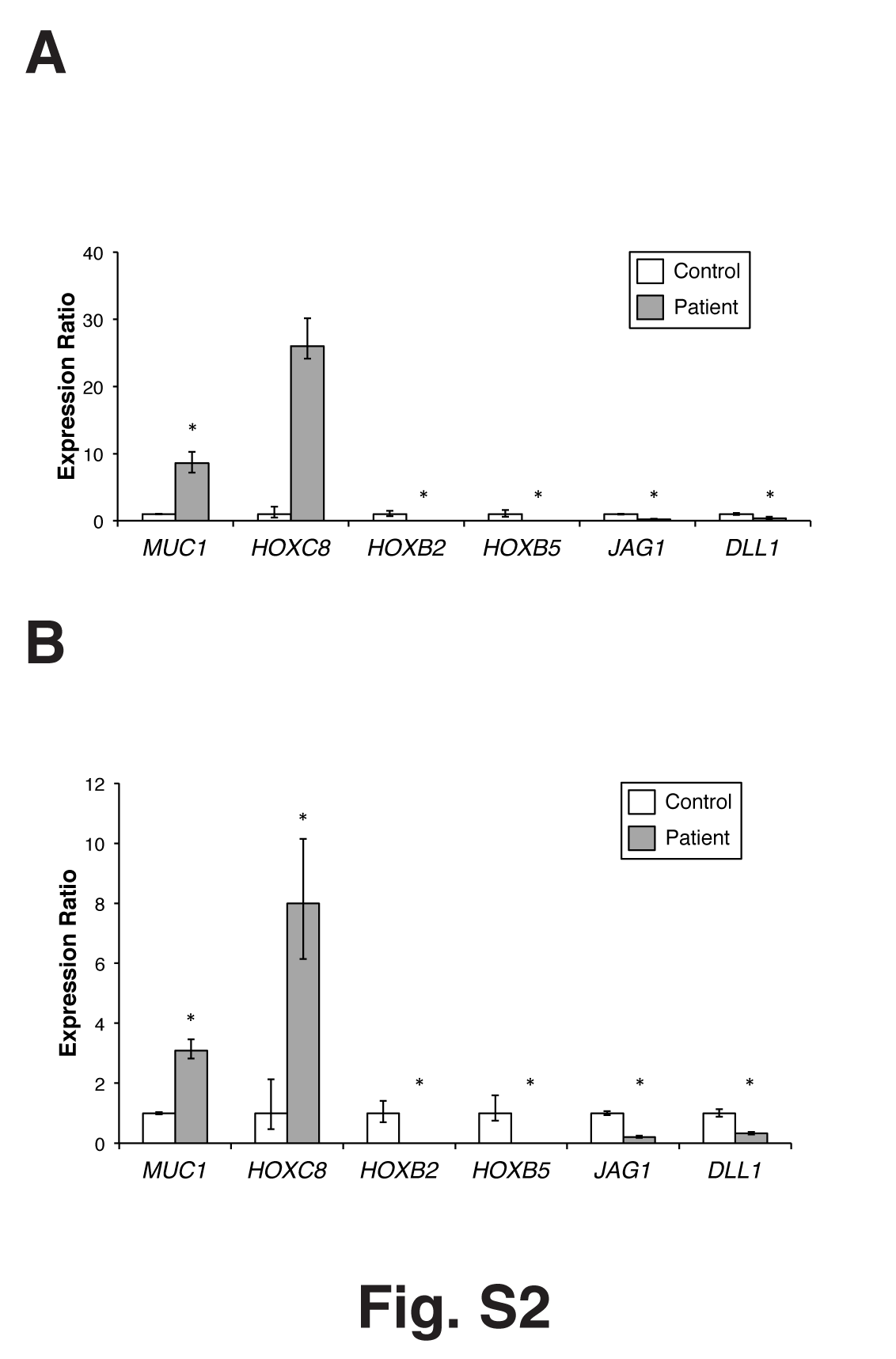

Supplement: Figure S2 — Comparison of expression levels of selected genes in vaginal mucosa and buccal mucosa of one MRKH patients. A) qRT-PCR analysis of mRNA expression levels of the six selected genes, in cell culture from vaginal mucosa of one MRKHS patient. For each gene, relative mRNA levels of patients are shown as fold value of the levels of one healthy subject (control). Each experiment was performed in triplicate, and mRNA levels were normalized to GAPDH mRNA expression. Error bars represent standard deviations (*P<0.01). B) qRT-PCR analysis of mRNA expression levels of the six selected genes in cell culture from buccal mucosa of one MRKHS patient. For each gene, relative mRNA levels of patient are shown as fold value of the levels of one healthy subject (control). Each experiment was performed in triplicate, and mRNA levels were normalized to GAPDH mRNA expression. Error bars represent standard deviations (*P<0.01). mRNA expression. Error bars represent standard deviations (*P<0.01). (TIF) [file pone.0091010.s002.tif]
