# Supplementary material for: Gene Expression Profile of Patients with Mayer-Rokitansky-Küster-Hauser Syndrome: New Insights into the Potential Role of Developmental Pathways
Source: PLoS One. 2014 Mar 7;9(3):e91010. doi: 10.1371/journal.pone.0091010 (PMC3946625; doi:10.1371/journal.pone.0091010)
Supplement: Data S1 — Supplementary Material and Methods. (DOC) [file pone.0091010.s003.doc]

**SUPPLEMENTARY MATERIAL AND METHODS**

**Immunofluorescence**

Human vaginal mucosa cells (HVMs), grown on coverslips, were processed for immunofluorescence as previously described [1] and incubated with cytokeratin 14 (K14) and 19 (K19) (1:100 in PBS; Santa Cruz Biotechnology, Santa Cruz CA, USA) antibodies. Primary antibodies were visualized using the appropriate FITC-conjugated IgG (1:100 in PBS; Jackson ImmunoResearch Laboratories, West Grove, PA, USA). Nuclei were visualized using 4’, 6-diamido-2-phenylindole dihydrochloride (DAPI) (1:10000 in PBS; Sigma-Aldrich, srl, Milano, Italy).

**Western blot analysis**

HVMs, HeLa, MCF-7 and human fibroblast were lysed in RIPA buffer. Total proteins (50 μg) were resolved under reducing conditions by 10% SDS–PAGE and transferred to Immobilon-FL membranes (Millipore). Membranes were incubated overnight at 4 °C with K14, K19 (1:200 dilution; Santa Cruz Biotechnology), or Vimentin (1:1000 dilution; Stemgent, CA, USA) followed by goat anti-mouse or goat anti-rabbit horseradish peroxidase (HRP)-conjugated secondary antibody (Sigma-Aldrich). Bound antibody was detected by enhanced chemiluminescence detection reagents (Pierce Biotechnology Inc, Rockford, IL, USA), according to manufacturer’s instructions.

**SUPPLEMENTARY REFERENCES**

1. Rotolo S, Ceccarelli S, Romano F, Frati L, Marchese C, Angeloni A. (2008) Silencing of keratinocyte growth factor receptor restores 5-fluorouracil and tamoxifen efficacy on responsive cancer cells. PLoS One 25: e2528.
